# Renal Ca^2+^ and Water Handling in Response to Calcium Sensing Receptor Signaling: Physiopathological Aspects and Role of CaSR-Regulated microRNAs

**DOI:** 10.3390/ijms20215341

**Published:** 2019-10-27

**Authors:** Marianna Ranieri

**Affiliations:** Department of Biosciences, Biotechnologies and Biopharmaceutics, University of Bari, 70125 Bari, Italy; marianna.ranieri@uniba.it; Tel.: +39-080-544-3334

**Keywords:** calcium sensing receptor (CaSR), calcium, microRNAs, aquaporin-2, kidney, renal water balance

## Abstract

Calcium (Ca^2+^) is a universal and vital intracellular messenger involved in a diverse range of cellular and biological processes. Changes in the concentration of extracellular Ca^2+^ can disrupt the normal cellular activities and the physiological function of these systems. The calcium sensing receptor (CaSR) is a unique G protein-coupled receptor (GPCR) activated by extracellular Ca^2+^ and by other physiological cations, aminoacids, and polyamines. CaSR is the main controller of the extracellular Ca^2+^ homeostatic system by regulating parathyroid hormone (PTH) secretion and, in turn, Ca^2+^ absorption and resorption. Recent advances highlight novel signaling pathways activated by CaSR signaling involving the regulation of microRNAs (miRNAs). miRNAs are naturally-occurring small non-coding RNAs that regulate post-transcriptional gene expression and are involved in several diseases. We previously described that high luminal Ca^2+^ in the renal collecting duct attenuates short-term vasopressin-induced aquaporin-2 (AQP2) trafficking through CaSR activation. Moreover, we demonstrated that CaSR signaling reduces AQP2 abundance via AQP2-targeting miRNA-137. This review summarizes the recent data related to CaSR-regulated miRNAs signaling pathways in the kidney.

## 1. Introduction

Calcium (Ca^2+^) is an universal and vital intracellular messenger involved in a diverse range of cellular and biological processes [1] extending from bone formation and neurotransmission to hormone secretion [2] and muscle contraction [3], and from gene expression [4] to cell proliferation [3,5]. The ability of a simple ion such as Ca^2+^ to play a pivotal role in cell biology results from the facility that cells have to shape Ca^2+^ signals in the dimensions of space, time, and amplitude. To generate the variety of observed Ca^2+^ signals, different cell types employ components selected from a Ca^2+^ signaling equipment, including an array of signaling, homeostatic, and sensory mechanisms. Changes in the concentration of (extracellular and intracellular) Ca^2+^ can disrupt the normal cellular activities and disrupt physiological function of these systems [6].

**Figure 1:**
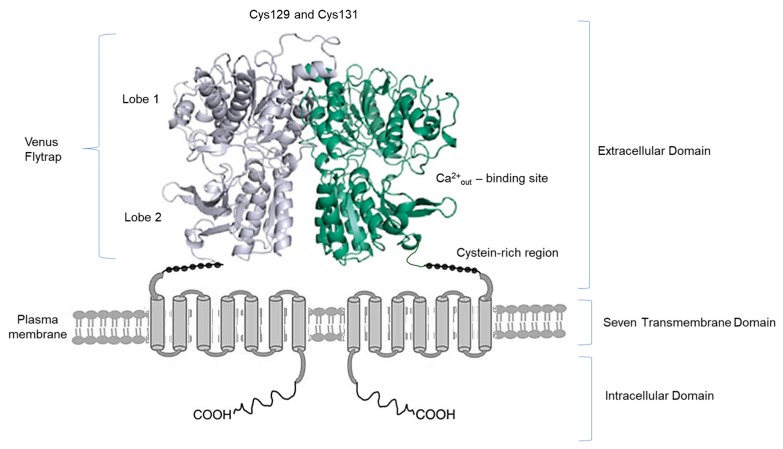
Schematic representation of the dimeric CaSR. Schematic representation of the CaSR homodimer structure at the plasma membrane, showing intracellular, transmembrane and extracellular domains, and Ca^2+^ binding site.

The extracellular calcium sensing receptor (CaSR) plays a critical role in regulating extracellular Ca^2+^ concentrations and cellular responses to these variations [7]. CaSR is a G-protein-coupled receptor discovered in 1993 by Brown and coworkers [7]. It comprises 612 amino acids and is followed by a 250 amino acid domain of seven transmembrane helices (TMD) and, finally, by a carboxy terminal (C) tail of approximately 200 amino acids [8,9] (Figure 1).

It is highly expressed in the parathyroid glands, where it regulates the production and secretion of parathyroid hormone (PTH) in a negative feedback manner. It is also expressed in numerous other tissues, where it has different but less well-defined functions. A key role of CaSR is to maintain extracellular Ca^2+^ levels by its ability to regulate PTH biosynthesis and release [10]. Moreover, CaSR also plays a central role in the control of the responsiveness of other target cells to Ca^2+^. This last function of CaSR assumes a considerable importance for renal reabsorption of Ca^2+^ and other essential ions [11,12], which will be better discussed in the following paragraphs.

The primary ligand for the CaSR is represented by extracellular Ca^2+^ [13]. Changes of this ion in the tissue spaces cause the activation of the receptor, even though CaSR is a promiscuous receptor activated by orthosteric agonists and allosteric modulators. One of the most curious features of CaSR is its pleiotropicity; in fact, different endogenous ligands are able to activate the receptor, which, in turn, initiates multiple intracellular pathways, also in the same cell type or the same signaling in the different body districts.

CaSR was found to be expressed in all of the key tissues that participate in extracellular Ca^2+^ homeostasis, including parathyroid cells, the thyroidal calcitonin-secreting C-cells [14], kidney [15], bone [16], and intestine [17]. Several studies have shown that the CaSR is also expressed in many other tissues in the body not playing obvious role in extracellular Ca^2+^ homeostasis, such as the breast, blood vessels, liver, airways, and various regions of the brain, where physiological roles of CaSR are not well understood.

Given the critical role of the CaSR in the regulation of the entire extracellular Ca^2+^ homeostatic system, few alterations in CaSR functionality, in its molecular partners and in signaling pathways, are expected to significantly imbalance mineral metabolism. In fact, CaSR deletion in mice, CaSR mutations in humans, and the use of negative and positive allosteric modulators have enabled the identification of activating or inactivating CaSR mutations or polymorphisms that cause significant alterations in calcium metabolism. The most important ones are those that cause familial hypocalciuric hypercalcemia (FHH), neonatal severe hyperparathyroidism, or autosomal dominant hypocalcemia (ADH), in which loss-of-function mutations in CaSR (for instance, CaSR-R392X) promote disruption of the downregulation mechanisms of PTH secretion [18].

On the other hand, the gain-of-function mutations result in the hypersensitivity of the CaSR causing hypocalcaemia due to a premature inhibition of PTH secretion. In particular, gain-of-function CaSR mutations result in autosomal dominant hypocalcemia (ADH) or type 5 Bartter syndrome and eight of them are clustered in loop 2 close to the two cysteines responsible for receptor homodimerization, i.e., cys129 and cys131 [19]. Recently, we characterized two different gain-of-function mutations of CaSR [20], N124K and R990G. While the N124K CaSR mutations causing ADH in humans are located in the extracellular domain, the intracellular tail of the receptor has one non-conservative polymorphism, R990G, which also confers a gain-of-function to the receptor (lower external calcium EC50) and in humans is associated with primary hypercalciuria in patients [21,22,23]. Although the two gain-of-function CaSR variants examined regard modifications at opposite locations within the CaSR protein sequence, their functional analysis revealed comparable biological regulatory effects within cells (significantly higher calcium accumulation in the Endoplasmic Reticulum (ER) and Sarco-Endoplasmic Reticulum Calcium ATPase (SERCA) expression and activity and reduced expression of the Plasma-Membrane Calcium ATPase (PMCA), which is perfectly in line with the comparable (low) basal cytosolic Ca^2+^ concentration found in cells expressing hCaSR-Wt) [20].

CaSR activation has also been proven to play a positive role in correcting some alterations found in cell models of Autosomal Dominant Polycystic Kidney Disease (ADPKD). Specifically, we recently demonstrated that in human conditionally immortalized proximal tubular epithelial cells silenced for *PKD1* (ciPTEC-PC1KD) or generated from a patient with ADPKD1 (ciPTEC-PC1Pt), selective activation of the CaSR increases cytosolic Ca^2+^, reduces intracellular cAMP and mTOR activity [24], and rescues defective ATP mitochondrial production [25], reversing the principal ADPKD dysregulations.

Additionally, it has been shown that CaSR is expressed in several cell types in the cardiovascular system, including endothelium, vascular smooth muscle cells (VSMC), and even in the perivascular nerve [26]. In this system it has been demonstrated that CaSR activation in endothelial cells had a hypotensive effect [27]. Additionally, Schepelmann et al. recently showed that a mouse model of targeted CaSR deletion from VSMC displayed reduced endothelium contractility in the aorta and mesenteric artery compared to wild-type animals in response to different stimuli [28,29]. Finally, in 2009 Romani et al. provided evidence that cardiac microvascular endothelial cells (CMEC) express CaSR, which is able to respond to physiological agonists by mobilizing Ca^2+^ from intracellular InsP3- sensitive stores [30].

CaSR may also be involved in another dangerous pathology affecting the cardiovascular system: the vascular calcification, a common complication of chronic kidney disease (CKD). In 2015, Molostvov et al. showed that in vitro treatment with calcimimetics reduces calcification of VSMC, supporting a role for CaSR in vascular calcification [31].

More recently, the CaSR has emerged as a potential therapeutic target for asthma [32]. The effects of calcilytics on the release of amyloid β peptides in cells treated with amyloid β surrogates have suggested the involvement of CaSR in Alzheimer’s Disease (AD) [33,34,35]. In addition, new and recent data highlighted the role of CaSR in cancer [36,37,38].

The central topic of this review is mainly focused on renal Ca^2+^ handling and on renal CaSR activation and signaling.

## 2. Ca^2+^ Handling and CaSR in the Kidney

The kidney is the major regulator organ of calcium and water homeostasis in the body. To carry out this important function, the kidney must be able to sense, detect, and respond to changes in its environment. Toka, Pollak, and Houillier define the kidney as a “*calcium-sensing organ*” because changes in extracellular fluid calcium concentration can affect the renal handling of various ions and water, independently of changes in PTH level [39].

Calcium handling through the small intestine wherein calcium absorption occurs, the bone wherein calcium is stored, and the kidney wherein absorbed calcium is eliminated, are fine controlled and regulated by a large number of transport mechanisms, hormones, and complex feedback systems. These control mechanisms are extremely important to prevent biomineralization processes in tissues where calcification is not a physiological event.

In plasma, a concentration of ~2.5 mmol/L for calcium and 1 mmol/L for phosphate may cause spontaneous phenomena of crystallization, if inhibitors of calcification, such as magnesium, fetuin A, osteoprotegerin, or matrix gla protein are not present. Likewise, in urine, calcium has a concentration of ~3 mmol/L and phosphate 10 mmol/L; in this contest, calciuria appears to be a major promoter of urine crystallization and stone formation. Hypercalciuria contributes to Randall’s plaque formation and to kidney stone formation [40,41,42,43].

As revised by Moor and Bonny in 2016 [44], in the proximal tubule, calcium is mainly reabsorbed paracellularly, partially driven by the activity of the sodium/proton exchanger (NHE3). In the thick ascending limb, calcium is reabsorbed by specialized and finely-controlled paracellular pathways involving claudins (16, 19, and 14) and the driving force is provided by sodium reabsorption through the sodium/potassium/chloride cotransporter (NKCC2). Increased interstitial calcium concentrations activate the basolateral CaSR, which reduces NKCC2 activity and directly modulates paracellular calcium permeability. In the distal convoluted and connecting tubules, calcium enters in cells through the apical transient receptor potential cation channel subfamily V member 5 (TRPV5 channel), binds intracellular calbindin, and exits through the basolateral sodium/calcium exchange (NCX1) and calcium ATPase (PMCA4). At the collecting duct level calcium is not reabsorbed. However, urine calcium variations are sensed by apical CaSR, resulting in the inhibition of water reabsorption [45] and in the stimulation of urine acidification [46,47]. Both of these mechanisms reduce calcium salt precipitation and, consequently, the risk of stone formation [48]. In line, calcilytics are indicated as a novel, promising avenue for the treatment of hypercalciuria, nephrolithiasis, and nephrocalcinosis [12].

As already mentioned, the kidney is a key organ for calcium homeostasis, and its ability to sense extracellular calcium levels in the urinary filtrate and interstitial fluid is due to the CaSR, which is expressed in several sites along the nephron [12,13,49,50]. Moreover, changes in extracellular calcium concentration affect several functions of the renal tubule, for instance, the water homeostasis. While CaSR expression in the thick ascending limb is commonly accepted, in other tubular and glomerular cells of the kidney it remains a subject of debate [51].

Riccardi and Valenti recently (2016) revised the localization and function of CaSR in the renal district [12]. CaSR is expressed across the entire length of the nephron, with the highest expression on the basolateral membrane of thick ascending limb epithelial cells where it plays a crucial role in the regulation of divalent mineral cation transport by inhibiting calcium reabsorption in response to a stimulation by an increase in plasma calcium levels [13,50,52,53,54]. In the proximal tubule, the CaSR is expressed apically and, here, rapidly and directly blunts the phosphaturic action on PTH, modulating the inhibitory action of PTH on Pi absorption [55]. In the collecting duct principal cells, CaSR is co-expressed with aquaporin-2 (AQP2) on the apical membrane [45,49,56], where it senses extracellular (urinary) Ca^2+^ and regulates the water reabsorption in order to control hypercalciuria and prevent Ca^2+^ crystal formation.

**Figure 2:**
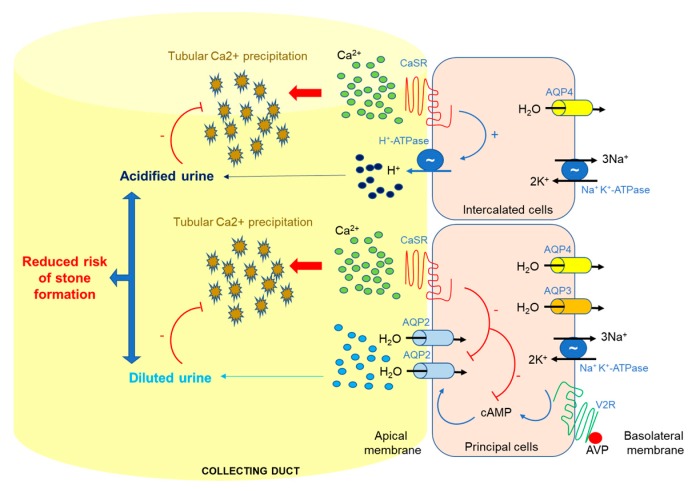
Localization and function of CaSR in the collecting duct. In the presence of an antidiuretic stimulus, Ca^2+^ concentrations in the pre-urine can become super-saturating, potentially leading to Ca^2+^ stone formation. When the luminal Ca^2+^ concentration becomes critically high it activates the apical CaSR which, in principal cells, blunts vasopressin-mediated apical insertion of the aquaporin-2 (AQP2) water channel and the rate of water reabsorption. In intercalated cells, CaSR activation leads to luminal acidification. Overall, these two effects result in the production of a dilute, acidified urine, which reduces the risk of nephrolithiasis.

In the intercalated cells of the collecting duct, CaSR is co-expressed with vacuolar type H+-ATPase at the apical membrane. When the luminal Ca^2+^ concentration becomes critically high, it activates the apical CaSR which, in principal cells, blunts vasopressin-mediated apical insertion of the AQP2 water channel and the rate of water reabsorption. In intercalated cells, CaSR activation leads to luminal acidification by activation of the H+-ATPase (Figure 2). These two effects together result in the production of a dilute, acidified urine, which reduces the risk of nephrolithiasis [12,57].

## 3. CaSR and AQP2 Interplay

A postulated mechanism for the process occurring in the collecting duct is that, during the vasopressin antidiuretic action promoting water reabsorption from the lumen, urinary Ca^2+^ concentration increases secondary to urine concentration. Increased Ca^2+^ levels, in turn, activate the CaSR located on the apical membrane of the principal cells. CaSR activation reduces the vasopressin-stimulated insertion of AQP2 into the plasma membrane and the rate of water reabsorption, consequently reducing the risk of Ca^2+^ supersaturation [58,59,60,61]. Maintenance and regulation of water balance is essential for all physiological processes and is critically dependent on water intake and water output in the kidney under the control of the antidiuretic hormone vasopressin. Dysregulation associated with water balance is responsible of several disorders, such as congenital nephrogenic diabetes insipidus (NDI), idiopathic syndrome of inappropriate antidiuretic hormone secretion (SIADH), nephrogenic syndrome of inappropriate antidiuresis (NSIAD), and autosomal dominant polycystic kidney disease (ADPKD) (revised in Ranieri et al., 2019) [62].

Already in the 1997, Sands and coworkers reported evidence of the presence of an apical *“Calcium/polycation receptor proteins (CaRs)”* in rat kidney terminal inner medullary collecting duct (tIMCD) that specifically reduces vasopressin-elicited osmotic water permeability when luminal calcium rises. This evidence provides support for a unique and new tIMCD apical membrane signaling mechanism linking calcium and water metabolism [45].

However, clinical evidence for an effect of luminal calcium on AQP2-mediated water reabsorption was provided for the first time, in humans (enuretic children), in a study of Valenti and collaborators, demonstrating that urinary AQP2 and calciuria correlate with the severity of enuresis [63]. Interestingly, hypercalciuric enuretic children receiving a low calcium diet to reduce hypercalciuria, had decreased overnight urine output (reduced nocturnal enuresis) paralleled by an increase in nighttime AQP2 excretion and osmolality [63]. Further evidence has been provided, more recently, in a bed rest study. Immobilization results in alterations of renal function, fluid redistribution, and bone loss, which couples to a rise of urinary calcium excretion. Under these conditions it was observed that bed rest induced an increase in blood hematocrit (reflecting water loss) which coincided with a reduction of urinary AQP2 likely paralleled by an increase in urinary calcium due to bone demineralization [64].

All these results strongly support the indication that urinary calcium can modulate the vasopressin-dependent urine concentration through a down-regulation of AQP2 trafficking.

In a previous study, we demonstrated that in cultured renal cells and microdissected collecting ducts, the inhibitory effect of CaSR signaling on AQP2 trafficking to the plasma membrane is associated with a significant decrease in cAMP-induced AQP2 phosphorylation at serine 256 (pS256) and AQP2 trafficking, resulting in a reduced osmotic water permeability response [65]. Specifically, calcimimetics activation of CaSR reduced AQP2 translocation to the plasma membrane in response to the cAMP elevation forskolin-induced. These data were also confirmed in HEK-293 cells transfected with two gain-of-function variants of CaSR, the CaSR-N124K mutation and the CaSR-R990G polymorphism, exploited to mimic “tonic” activation of CaSR [20]. The physiological consequence of the negative feedback on cAMP-induced AQP2-pS256 phosphorylation and trafficking stimulated by CaSR signaling is lowering the osmotic water permeability response both in cells and in isolated mouse collecting duct [65].

This theory that elevated concentration of calcium in urine counteract vasopressin action via the activation of CaSR expressed at luminal membrane of principal cells has been further validated in a mouse model double-knockout (dKO) for Pendrin/NaCl Cotransporter (NCC) [66], which display significant calcium wasting and severe volume depletion, despite high circulating vasopressin levels [67].

Due to severe hypercalciuria, a tonic activation of the luminal CaSR in the collecting duct is expected in this dKO mice model and, quite interestingly, those mice had a strong reduction in total AQP2 expression associated with a significantly higher expression of AQP2-pS261 and ubiquitinated AQP2. In addition, in dKO mice, exposure of inner medulla kidney slices to the proteasome inhibitor MG132 increased total AQP2 by 50%, indicating that the rate of AQP2 degradation via proteasome is significantly higher. It has been recently suggested that CaSR expressed at the apical membrane of collecting duct principal cells could mediate the effects of hypercalciuria in reducing vasopressin-elicited osmotic water permeability and urinary concentrating ability by the activation of autophagic degradation of AQP2. Indeed, proteomic analysis of inner medullary collecting ducts isolated from parathyroid hormone-treated rats revealed increased autophagic degradation of a specific set of proteins including AQP2 [68].

Interestingly, the functional link between CaSR and AQP2 degradation was supported by the observation that the reduced total AQP2 and higher levels of AQP2-pS261 found in dKO mice are paralleled by higher levels of p38 mitogen-activated protein kinase (p38-MAPK), an enzyme activated by CaSR signaling and known to phosphorylate AQP2 at Ser261 [69,70]. Of note, CaSR inhibition with the calcilytic NPS2143 reduced AQP2-pS261 levels in dKO mice, demonstrating that CaSR acts upstream of p38-MAPK and mediates the upregulation of AQP2-pS261. Moreover, inhibition of p38-MAPK caused a drastic decrease in AQP2-pS261, along with a nearly five-fold increase in total AQP2. Furthermore, in dKO mice, p38-MAPK inhibition results in a drastic reduction in ubiquitinated AQP2 that is paralleled by a strong increase in total AQP2 [66].

In addition to the effect on AQP2 trafficking, previous findings demonstrated that high external calcium reduces AQP2 expression both in the collecting duct cell line mpkCCD and in hypercalciuric rats [71,72]. Moreover, vitamin D-elicited hypercalcemia/hypercalciuria is associated with polyuria in humans. At the end, dihydrotachysterol (DHT) induces AQP2 water channel downregulation despite unaltered AQP2 mRNA expression in rats, suggesting a higher rate of AQP2 degradation attributed to activation of the calcium-sensitive protease calpain [73].

Ultimately, these data support a direct effect of luminal calcium on AQP2 expression in collecting duct principal cells and point to a role of calcium in regulating both AQP2 trafficking and expression.

Of note, regulation events of post-transcriptional gene expression can occur and be involved in several diseases, under the direct control of the small non-coding RNAs, the microRNAs (miRNAs) [74].

## 4. CaSR-Regulated miRNAs

MiRNAs are ubiquitous endogenous, short non-coding, most frequently of 19–25 nucleotides in length, single-stranded (ss)RNA transcripts that act as post-transcriptional regulators of gene expression by blocking protein translation and/or inducing messenger RNA (mRNA) degradation. miRNAs may act as transcriptional or splicing regulators within the nucleus [74], and be involved in genetic exchange with adjacent cells, through exosomes [75]. Many miRNAs display tissue-specific expression patterns and are involved in the development and maintenance of organ function. Approximately 60% of protein-coding genes are influenced by miRNAs [76] that play crucial roles in several biological processes, including control of cell cycle and differentiation, proliferation, and metabolism. As such, miRNA deregulation is being increasingly associated with several human pathologies [77]. Since their discovery in 1993 [78], numerous miRNAs have been identified in humans and other eukaryotic organisms, and their role as key regulators of gene expression is still being elucidated.

Only since 2012 have the miRNA activated by CaSR been indicated as key regulators of diverse proteins involved in different pathophysiological circumstances. Indeed, Hou and co-workers described the physiological function of claudins in the paracellular transport mechanisms with a focus on renal Ca^2+^ handling [79] (revised also in 2016 in [80]). In the thick ascending limb of Henle, paracellular Ca^2+^ reabsorption involves the functional interplay of three important claudin genes: claudin-14, -16, and -19, associated with human kidney diseases with hypercalciuria, nephrolithiasis, and bone mineral loss. A novel microRNA-based signaling pathway downstream of CaSR that directly regulates claudin-14 gene expression has been described indicating that claudin-14 is a key regulator for renal Ca^2+^ homeostasis. Through physical interaction, claudin-14 blocks the paracellular cation channel made of claudin-16 and -19, critical for Ca^2+^ reabsorption in the tick ascending limb. The molecular cascade of CaSR-microRNAs-claudins forms a regulatory loop to maintain proper Ca^2+^ homeostasis in the kidney [79,81]. Under normal dietary condition, claudin-14 proteins are suppressed by two microRNA molecules, miR-9 and miR-374. Both microRNAs directly target the 3′-UTR of claudin-14 mRNA; induce its mRNA decay and translational repression in a synergistic manner, causing claudin-14 to decline, leading to decreases in cation permeation [81,82,83,84]. These data indicate that the regulation of miRNA by CaSR signaling may occur on several layers within the kidney.

Moreover, the silencing of the CaSR has been demonstrated to induce tumors in colorectal cancer, associated with increased expression of miR135b and miR-146b, which are considered to be oncogenic [85]. In colon cancer cell lines other miRNAs—miR21, miR-145, and miR-135a—are inversely correlated with CaSR expression [86,87].

Furthermore, altered expression of miRNAs have been implicated in parathyroid function and may have an important role in the development of parathyroid tumors [88,89].

Our recent studies suggest that CaSR may regulate AQP2 expression also via miRNA [66] (Table 1).

However, despite several studies having demonstrated that transcriptional and post-transcriptional regulation of AQP2 play crucial roles in AQP2 expression levels within the cell, along with a profound impact on water homeostasis [90,91], little is known about the role of miRNA in the regulation of AQP2 expression.

Several studies highlight an emerging role of miRNAs in AQP regulation (reviewed in [92]). Specifically, miRNAs have been identified as endogenous modulators of the expression of several AQPs [93,94,95,96,97,98,99,100,101,102,103]. Two AQP2-targeting miRNAs, miR-32 and miR-137, were reported to decrease AQP2 expression in kidney collecting duct cells independently of vasopressin regulation [96]. The authors demonstrated a significant decrease of AQP2 translation in mpkCCDc14 cells transfected with miR-32 or miR-137 providing novel insights into the regulation of AQP2 by RNA interference.

Of interest, we have recently correlated the AQP2-targeting miR-137 with the reduced expression of AQP2 in a Pendrin/NaCl cotransporter dKO mouse model, a mechanism found to be mediated by CaSR signaling [66]. Specifically, in dKO mice, miR-137 was found to be about 1.7-fold higher compared to WT mice, which was in line with the reduced translation of AQP2 mRNA. Noteworthy, miR-137 transcript levels were increased by the calcimimetic NPS-R-568 in WT mice; furthermore, in dKO mice, miR-137 transcript levels were drastically reduced in response to CaSR or p38-MAPK inhibition with the calcilytic NPS2143 or SB203580, respectively, providing the first evidence that CaSR signaling directly acts upstream of the miR-137-AQP2 axis [66].

These findings represent the first demonstration that CaSR can regulate AQP2 expression via AQP2-targeting miRNA.

The discovery of miRNAs as endogenous modulators of AQPs offers a potential therapeutic approach for the regulation of AQP-related disorders [92].

## 5. Regulation of miRNA Expression and Therapeutic Perspectives

MiRNAs participate in numerous cellular regulatory pathways.

Emerging miRNA studies show promise for these transcripts as both biomarkers and as therapies in cancer, and cardiovascular and renal pathologies [104].

Quantitative and qualitative assessment of miRNA expression have clearly shown consistent changes in miRNA expression profiles in various diseases. Thus, profiling of miRNA expression can be an important tool for diagnostics and treatment of disease.

Distinct mechanisms of regulation of miRNA expression can occur in cell: transcriptional (changes in gene expression and promoter hypermethylation) and post-transcriptional (changes in miRNA processing), as well as effects of endogenous (hormones, cytokines) and exogenous (xenobiotics) compounds on the miRNA expression (reviewed in [105,106]).

The great interest for miRNAs as a novel class of functional regulators of tissue maintenance and stress responses requires appropriate and reliable identification tools and suitable techniques for measuring and modulating microRNAs in different model systems.

Recently, several strategies for gain- and loss-of-function studies for specific miRNAs both in vitro and in vivo have been developed. For instance, in vitro miRNA manipulation consists in transient transfections of miRNA mimics or miRNA antagonists [107]. Another refined way to study the functional relevance of a miRNA is by genetic deletion. In fact, numerous examples of miRNA knockout animals have been published and have revealed specific functions for the deleted miRNAs, particularly under illness conditions [108,109,110]. For in vivo studies, there are several tools available to selectively target miRNA pathways. The most used approach is by using antimiRs. AntimiRs are modified antisense oligonucleotides that can reduce the endogenous levels of a miRNA, resulting in an increase of expression of target genes.

In addition to the antimiRs, there is also the opportunity to mimic or re-express miRNAs by using synthetic RNA duplexes designed to mimic the endogenous functions of the miRNA of interest [107,111].

The relative fluency by which miRNAs can be manipulated pharmacologically provides interesting therapeutic opportunities.

In the kidney, the possibility of using miRNA alone or in combination with other drugs to specifically modulate the vasopressin–AQP2 pathway, provides new cues for therapies that target AQP2 [7]. In this respect, the recent identification by Bijkerk and co-workers of miR-132 as a first miRNA target which post-transcriptionally blocks vasopressin gene expression regulating the osmo-balance has opened new avenues for drug development [112].

## 6. Conclusions

Although for cellular and molecular physiology the prominence of miRNAs results clear and explicit, however the signaling pathways that activate them still remain to be understood. In the kidney, CaSR-regulated miRNAs and the effect on gene expression of proteins playing key roles in the Ca^2+^ handling are still largely unexplored.

**Figure 3:**
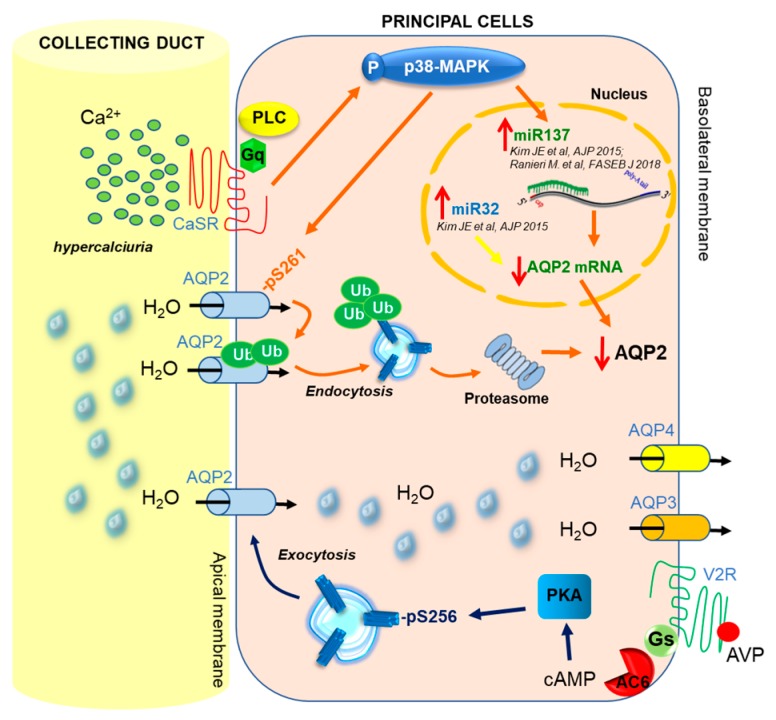
Schematic model. The proposed model shows that high urinary calcium levels in the collecting duct activate CaSR, resulting in the phosphorylation and activation of p38-MAPK that, in turn, phosphorylates AQP2 at Ser261, causing AQP2 internalization, ubiquitination (Ub), and proteasomal degradation. In parallel, CaSR signaling promotes the synthesis of miRNA-137 (orange arrows) via the activation of p38-MAPK, which results in reduced AQP2 mRNA translation (described in Ranieri et al., 2018 [66]). As reported by Kim et al. 2015 [96], also miRNA-32 (yellow arrow) is able to reduce AQP2 mRNA levels and consequently AQP2 abundance, however, the precise signaling pathway through which these events occur are still unknown.

In this context, the identification of a novel physiological mechanism that, in the inner medulla, links the activation of the CaSR by high urine Ca^2+^ levels to the regulation of the AQP2 expression and trafficking open new avenues for understanding AQP2 regulation and, consequently, how water balance is influenced by hypercalciuria (see schematic model in Figure 3).

## Figures and Tables

**Table 1:** MicroRNA expression downstream CaSR signaling.

miRNA		Target mRNA	Target Protein	Target Organ	References
**miR-9** **miR-374**	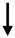	*CLDN14*	Claudin-14	Thick Ascending Limb cell, kidney	Hou J, Organogenesis 2012 [79]; Gong Y, Hou J, JASN 2014 [82]; Gong Y et al., JASN 2015 [83]; Hou J, Curr Opin Nephrol Hypert 2016 [80]
**miR-21** **miR-135a** **miR-135b**	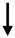	Tumor suppressors	Tumor suppressor proteins	Human colon carcinoma cell lines, colon	Singh N et al., Int J Cancer, 2013 [86,87]
**miR-145**	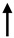	Oncogenes	Oncoproteins	Human colon carcinoma cell lines, colon	Singh N et al., Int J Cancer, 2013 [86,87]
**miR-375** **miR-429** **miR-361**	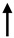	*PTH*	Parathormone	Parathyroid	Shilo V et al., FASEB J 2015 [88]
**miR-137**	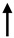	*AQP2*	Aquaporin-2	Collecting duct, kidney	Ranieri M et al., FASEB J 2018 [66]
